# Activity of ceritinib in crizotinib-resistant ROS1-rearranged non-small-cell lung cancer patients

**DOI:** 10.1097/MD.0000000000033543

**Published:** 2023-07-21

**Authors:** Huixian Zhang, Xingya Li, Ziheng Zhang, Siyuan Huang, Qianqian Guo, Ningning Yan

**Affiliations:** a Department of Medical Oncology, The First Affiliated Hospital of Zhengzhou University, Zhengzhou City, Henan Province 450052, People’s Republic of China.

**Keywords:** ceritinib, crizotinib-resistant, NSCLC, ROS1

## Abstract

As a second-generation selective oral anaplastic lymphoma kinase inhibitor, ceritinib is an effective first-line treatment for c-ros oncogene 1 (ROS1)-rearranged non-small-cell lung cancer (NSCLC). Its efficacy and safety for the treatment of crizotinib-resistant ROS1-rearranged NSCLC were explored in the study. A retrospective single-center study was conducted to investigate the efficacy of ceritinib in crizotinib-resistant ROS1-rearranged NSCLC. The objective response rate was the primary objective, while the disease control rate, progression-free survival and adverse events were secondary objectives. From December 2015 to October 2021, a total of 246 patients with ROS1-rearranged NSCLC were screened, 12 (4.9%) of whom were treated with ceritinib after the development of crizotinib resistance. Among the 12 crizotinib-resistant patients included, 3 displayed the efficacy of partial response and 3 had the efficacy of stable condition. The objective response rate, disease control rate and median progression-free survival of all patients were 25% (95% confidence interval [CI]: −3.7% to 53.7%; 3 of 12 patients), 50% (95% CI: 16.8% to 83.2%; 6 of 12 patients), and 10.5 months (95% CI, 5.7 to 15.3 months), respectively. In addition, of the 6 patients with brain metastases, an intracranial disease control rate of 66.7% (95% CI:12.5% to 120.9%) was obtained. The research results reveal that ceritinib can be a treatment option for ROS1-rearranged NSCLC patients after the development of crizotinib resistance.

## 1. Introduction

The c-ros oncogene 1 (ROS1) rearrangement is a distinct molecular subset of various cancers, occurring in approximately 1% to 2% of patients with non-small-cell lung cancer (NSCLC).^[1–3]^ Reportedly, lung adenocarcinoma patients who are young, nonsmokers, and Asians are subject to a higher incidence of ROS1 rearrangement.^[4]^ The ROS1 rearrangement partners include but are not limited to CD74, CCDC6, EZR, FIG, and KDELR2.^[5]^ Because ROS1 and anaplastic lymphoma kinase (ALK) are structurally similar and share 49% amino acid sequence homology in their kinase domains, ROS1-positive NSCLC is generally sensitive to ALK inhibitors.^[6]^ Crizotinib, an inhibitor of Mesenchymal epithelial transforming factor/ALK/ROS1 tyrosine kinase (TKI), was the first inhibitor approved by the US Food and Drug Administration in 2016 for the treatment of ROS1-positive NSCLC.^[7,8]^ Crizotinib is effective in the initial stage but is eventually resisted by most patients.^[9]^ In addition, its low blood-brain barrier penetration leads to a high risk of brain recurrence.^[10,11]^

Ceritinib is a second-generation selective oral ALK inhibitor and proves to be effective in the first-line treatment for ROS1-positive NSCLC.^[12,13]^ It is recommended in the NCCN guidelines for the treatment of ROS1-positive metastatic NSCLC, based on an open-label, multicenter, phase II study.^[14]^ This study confirmed the effectiveness of ceritinib in ROS1-positive NSCLC patients who were not treated with crizotinib. In addition, the brain-blood contact rate of ceritinib crossing the blood-brain barrier was found to be approximately 15%.^[15]^ A high rate of intracranial response in patients with asymptomatic brain metastases was also identified. However, due to the inclusion of only 2 crizotinib-resistant patients, the efficacy of ceritinib in crizotinib-resistant ROS1-rearranged NSCLC patients remains unknown.

Given financial considerations, crizotinib is the first choice for most Chinese patients.^[16]^ However, no approved second-line targeted therapies are available once resistance to crizotinib has developed among patients.^[17]^ As a second-generation ALK inhibitor, ceritinib can overcome crizotinib resistance in ALK-positive NSCLC patients, but its efficacy in the treatment of crizotinib-resistant ROS1- rearranged NSCLC patients remains unclear.^[13,18]^

In the present retrospective study, patients who developed the development of resistance to crizotinib and were then treated with ceritinib were included to investigate the activity of ceritinib in crizotinib-resistant ROS1-rearranged NSCLC patients.

## 2. Materials and methods

### 2.1. Patients and samples

In this retrospective study, ROS1-rearranged NSCLC patients who were treated with ceritinib after the development of crizotinib resistance at the First Affiliated Hospital of Zhengzhou University between January 2016 and December 2021 were enrolled. Their medical records were reviewed and the TNM stage was determined according to the International Association for the Study of Lung Cancer (version 8). This study was approved by the Ethics Review Board of the First Affiliated Hospital of Zhengzhou University. The need for written informed consent was waived owing to the retrospective design of the study.

### 2.2. Study design

A retrospective single-center study was conducted to investigate the activity of ceritinib in crizotinib-resistant ROS1-rearranged NSCLC patients with the primary objective of evaluating the objective response rate (ORR) and secondary objectives of evaluating the disease control rate (DCR), progression-free survival (PFS) and adverse events (AEs). PFS was defined as the time from the beginning of ceritinib treatment to the first documented tumor progression or death. Patients who had not progressed/died at the data cutoff in April 2022 were considered to be censored at the last follow-up. ORR was defined as the proportion of patients who experienced a complete or partial response to ceritinib therapy, according to RECIST 1.1. DCR represents the proportion of patients with complete response, partial response (PR) or stable condition (SD). Moreover, AEs were graded according to the Common Terminology Criteria for Adverse Events (CTCAE) (version 5.0).

### 2.3. Data collection and assessment

The data of this study were collected from clinical records on medical history.

### 2.4. Statistical analysis

The median PFS and the number of patients at risk were estimated using the Kaplan–Meier method and associated 95% confidence intervals (CIs). The confidence intervals of the ORR equal proportions were calculated using the Clopper-Pearson method. A Log-rank test was conducted to analyze the differences between survival curves, and univariate Cox proportional hazards regression analyses were performed to examine the impact of clinicopathological factors on PFS. All statistical analyses were conducted using the SPSS version 26.0 software (IBM Corp), where *P* value <.05 was considered statistically significant.

## 3. Results

From January 2016 to December 2021, a total of 246 patients with ROS1-positive NSCLC were screened out, 12 (4.9%) of whom were treated with ceritinib after the development of crizotinib resistance. The included patients received oral ceritinib at the recommended dose of 450 mg/day in continuous 28-day treatment cycles. These 12 patients were composed of 5 females and 7 males, with a median age of 54 years old at diagnosis and an age range of 38 to 70 years old. All the patients were in stage IV. The majority of patients (75%) were never-smokers, and all had adenocarcinoma histology. Among the 12 patients included in the study, 1 was diagnosed with ROS1 gene fusion by immunohistochemistry, 2 were detected by RT-PCR, and the other patients were detected by NGS. Clinicopathological characteristics of the patients are presented in Table 1.

**Table 1:** Baseline characteristics.

Characteristics	Patients (n = 12) (%)
Age median (range), yr	54 (38–70)
Sex	
Male	7 (58.3)
Female	5 (41.7)
Smoking status	
Never smoker	3 (25)
Former or current smokers	9 (75)
Histology	
Adenocarcinoma	12 (100)
Stage	
IV	12 (100)
Brain metastases	
Yes	6 (50)
No	6 (50)
ECOG PS score	
0/1	10 (83.3)
2	2 (16.7)

ECOG = Eastern Cooperative Oncology Group.

**Figure 1.**
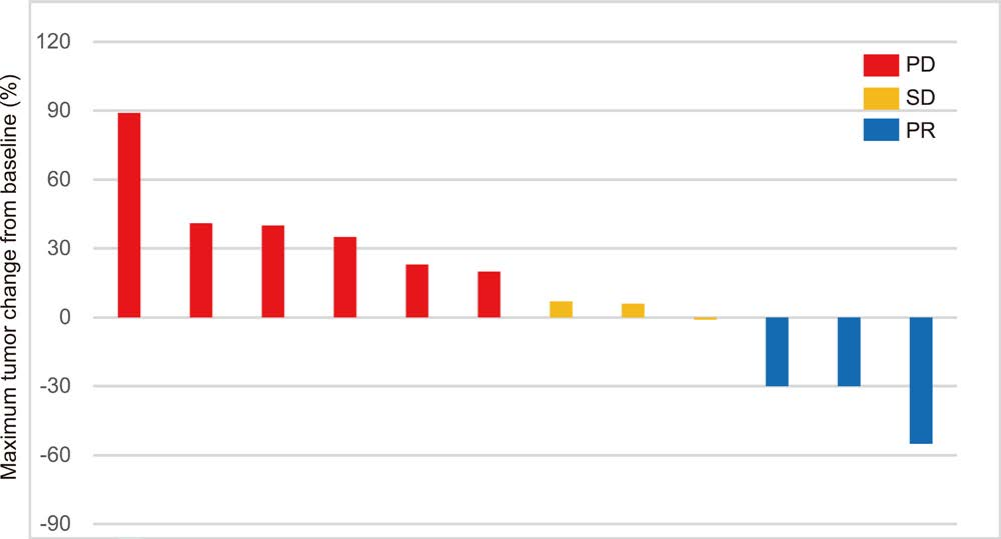
Best percentage change from baseline in tumor volume in patients with at least 1 postbaseline measurement. PD = progressive disease, PR = partial response, SD = stable disease.

**Figure 2.**
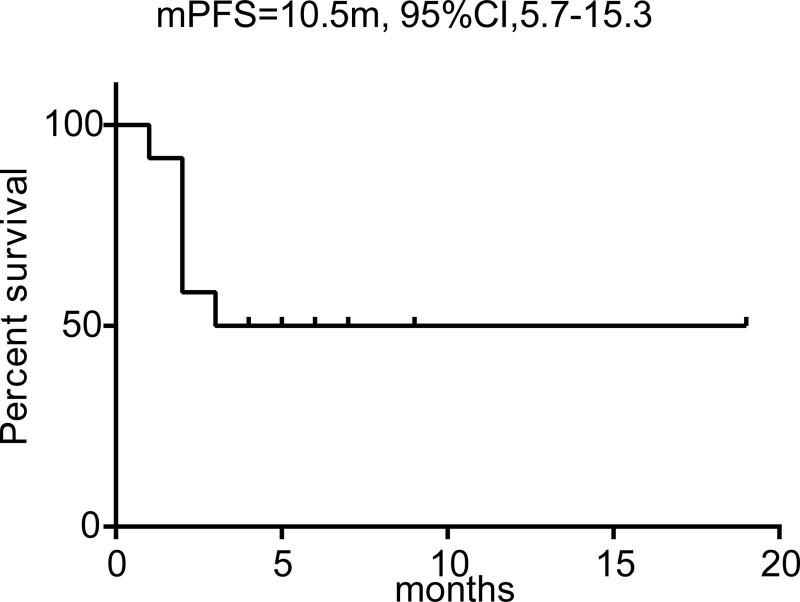
Kaplan–Meier curve of progression-free survival in all patients.

Among the 12 patients enrolled, 3 had efficacy assessments of PR and 3 had efficacy assessments of SD. Moreover, 6 patients progressed at the time of the first tumor evaluation. Overall, an ORR of 25% (95% CI: −3.7% to 53.7%; 3 of 12 patients) and DCR of 50% (95% CI: 16.8% to 83.2%; 6 of 12 patients) (Table 2) were obtained. A decrease in tumor burden from baseline was observed in 4 (33.3%) of the 12 patients (Fig. 1). Additionally, the median PFS was 10.5 months (95% CI, 5.7 to 15.3 months) (Fig. 2). According to univariate analyses, smoking status, brain metastases and sex were not significantly associated with PFS, however, the ECOG PS score was an independent prognostic indicator of PFS (hazard ratio (HR) 0.02, 95% CI 0.00–0.47, *P* = .02) (Table 3).

**Table 2:** Objective response to treatment.

Response	All patients, n	(%)
CR	0	0
PR	3	25
SD	3	25
PD	6	50
ORR	3	25 (95% CI: −3.7 to 53.7)
DCR	6	50 (95% CI: 16.8 to 83.2)

CI = confidence interval, CR = complete response, DCR = disease control rate, ORR = overall response rate, PD = progressive disease, PR = partial response, SD = stable disease.

**Table 3:** The correlation between clinicopathological characteristics and PFS.

	PFS		
Parameters	HR	95% CI	*P* value
Smoking status never vs former/current	5.998	0.83–43.1	.08
Brain metastases yes vs no	0.349	0.06–2.10	.25
PS 0/1vs2	0.02	0.00–0.47	.02*
Sex male vs female	0.19	0.03–1.21	.08

* *P* < .05.

CI = confidence interval, HR = hazard ratio, PFS = progression-free survival.

To assess intracranial response, intracranial lesions in patients with brain metastases were investigated (Table 4). At study entry, 6 patients had brain metastases, including 2 with PR, who had not received prior brain radiotherapy and presented a response time of 5 months and 7 months, respectively. In addition, 2 patients were assessed as having SD, and 2 other patients had intracranial lesion progression at the time of the first assessment. Overall, the ORR of intracranial lesions was 33.3% (95% CI: −20.9% to 87.5%) and disease control was achieved in 66.7% (95% CI: 12.5%–120.9%). Overall, the above intracranial results were consistent with systemic responses (Table 3).

**Table 4:** Best clinical response in cranial.

Response	Patients with brain metastases n = 6	%
CR	0	0
PR	2	33.3
SD	2	33.3
PD	2	33.3
ORR	2	33.3 (95% CI: −20.9 to 87.5)
DCR	4	66.7 (95% CI: 12.5 to 120.9)

CI = confidence interval, CR = complete response, DCR = disease control rate, ORR = overall response rate, PD = progressive disease, PR = partial response, SD = stable disease.

The incidence and types of AEs are summarized in Table 5. All 12 patients experienced at least 1 adverse event associated with ceritinib. As expected, gastrointestinal AEs were the most frequent. Notably, the incidences of nausea and vomiting were high (50% and 41.7%, respectively). In addition, 2 patients presented with rash, 1 with liver injury, 1 with vision disorder, and all with grades I to II. Four patients presented with grade 3 or higher toxicity, including nausea, vomiting, decreased appetite and constipation. The incidence of grade 3 and above vomiting was the highest, with severe vomiting occurring in 2 of the 12 patients (16.7%). All these AEs were relieved after symptomatic supportive treatment, and no ceritinib-related fatal AEs occurred.

**Table 5:** Adverse events.

Event	Treated population (12 patients have documented side effects)	
	Any grade	≥Grade 3
Nausea	6/12 (50%)	1/12 (8.3%)
Vomitting	5/12 (41.7%)	2/12 (16.7%)
Diarrhea	3/12 (25%)	0
Decreased appetite	2/12 (16.7%)	1/12 (8.3%)
Weight loss	2/12 (16.7%)	0
Constipation	3/12 (25%)	1/12 (8.3%)
Rash	2/12 (16.7%)	0
Liver injury	1/12 (16.7%)	0
Vison disorder	1/12 (16.7%)	0

## 4. Discussion

Both crizotinib and ceritinib are recommended by the NCCN guidelines as first-line treatment for patients with advanced ROS1-positive NSCLC.^[19]^ In East Asian patients with ROS1-positive advanced NSCLC, the ORR and mPFS after crizotinib treatment were 71.7% and 15.9 months, respectively.^[20]^Therefore, crizotinib remains the best first-line treatment option for such patients, especially for those from China, followed by the next generation of TKIs when the disease progresses.^[21]^ However, no approved second-line targeted therapies are available once resistance to crizotinib has developed.

Lorlatinib or brigatinib is effective in patients with crizotinib resistance as reported in a few studies, the sample sizes of which, however, are small.^[22–24]^ Lorlatinib is a highly brain-penetrant third-generation ALK and ROS1 inhibitor, which has been approved by the US Food and Drug Administration for the treatment of ALK-positive NSCLC.^[25]^ In a recent multicenter, single-arm, Phase I to II study, lorlatinib demonstrated efficacy in ROS1-positive patients previously treated with crizotinib with an ORR of 35% and mPFS of 13.8 months.^[22]^ Therefore, it has been recommended by NCCN guidelines as a follow-up option for ROS1-positive lung cancer with crizotinib or Entrectinib disease progression. Few studies have been conducted on the efficacy of the second-generation ALK inhibitor, brigatinib, for the treatment of ROS1-positive NSCLC after the development of crizotinib resistance. One retrospective study included a total of 7 patients, of whom 2 showed partial responses (ORR 29%) and 1 (14%) was in a stable condition.^[26]^

To the best of our knowledge, this is the first retrospective study to analyze the efficacy of ceritinib in the treatment of crizotinib-resistant ROS1-positive NSCLC patients. Among the 12 crizotinib-resistant patients included, 3 had an efficacy of PR and 3 had an efficacy of SD, with a disease control rate of 50%, an overall response rate of 25%, and a median PFS of 10.5 months. Because of the blood-brain barrier, the brain is considered a major pharmacological refuge. In fact, approximately 50% of the patients treated with ROS1-TKIs develop disease progression due to central nervous system metastases.^[17]^ A total of 6 patients with brain metastases were included in this study, 2 with a PR response to brain lesions. Overall, the ORR of intracranial lesions was 33.3% (95% CI: −20.9% to 87.5%) and intracranial disease control was achieved in 66.7% (95% CI: 12.5%–120.9%). The study results also indicated that ceritinib might have high activity in the central nervous system.

This study confirms that ceritinib is a treatment option for ROS1-positive NSCLC patients with crizotinib resistance. Apparently, intracranial and systemic activity is related to the underlying molecular mechanisms of crizotinib resistance. Two mechanisms of acquired crizotinib resistance have been reported, namely the ROS1-dependent mechanism and the ROS1-independent mechanism.^[27,28]^ Reportedly, mutations in the ROS1 kinase domain and activation of bypass signaling pathways are the most important mechanisms of crizotinib resistance among the identified mechanisms so far.^[29]^ In addition, approximately 50% to 60% of crizotinib resistance is caused by on-target mutations, of which G2032R is the most frequently observed mutation in ROS1-positive patients.^[30,31]^ Notably, ceritinib, entrectinib, and lorlatinib were found by preclinical studies to have no strong activity against G2032R.^[32]^ Also, in vitro studies have demonstrated that L2026M gatekeeper mutations maintain sensitivity to ceritinib and brigatinib.^[26]^ In this retrospective study, the crizotinib resistance pathway that might be more sensitive to ceritinib was not explored. This should be considered in future research.

This study is still subject to some limitations. First, all enrolled patients were from the same hospital, therefore the results could not represent the entire population. Second, this study was retrospective, and complete clinical information of the patients might not have been collected. Third, progressive and post-progressive biopsies, which are critical for understanding the mechanisms of response and/or resistance, are lacking. Moreover, the sample size was limited. Therefore, the results should be validated in prospective studies with larger sample sizes.

## 5. Conclusion

Based on a retrospective analysis of the efficacy of ceritinib in the treatment of ROS1-positive NSCLC patients after the development of crizotinib resistance, ceritinib can be used as a treatment option after the development of crizotinib resistance. However, further research is needed to find out how to screen ceritinib-sensitive patients.

## Acknowledgments

This study was supported by a grant of the Henan Provincial Science and Technology Research Project (Grant N. 2008020081).

## Author contributions

**Conceptualization:** Huixian Zhang, Xingya Li.

**Formal analysis:** Huixian Zhang, Xingya Li.

**Funding acquisition:** Huixian Zhang.

**Investigation:** Huixian Zhang, Ningning Yan.

**Methodology:** Huixian Zhang.

**Supervision:** Siyuan Huang.

**Software:** Ningning Yan.

**Validation:** Ziheng Zhang.

**Visualization:** Huixian Zhang, Qianqian Guo.

**Writing – original draft:** Huixian Zhang, Xingya Li, Ningning Yan.

**Writing – review & editing:** Huixian Zhang, Xingya Li, Ziheng Zhang.

## References

[1] Takeuchi K Soda M Togashi Y. RET, ROS1 and ALK fusions in lung cancer. Nat Med. 2012;18:378–81. 22327623 10.1038/nm.2658

[2] Skoulidis F Heymach JV. Co-occurring genomic alterations in non-small-cell lung cancer biology and therapy. Nat Rev Cancer. 2019;19:495–509. 31406302 10.1038/s41568-019-0179-8 PMC7043073

[3] Bergethon K Shaw AT Ou SH. ROS1 rearrangements define a unique molecular class of lung cancers. J Clin Oncol. 2012;30:863–70. 22215748 10.1200/JCO.2011.35.6345 PMC3295572

[4] Lin JJ Shaw AT. Recent advances in targeting ROS1 in lung cancer. J Thorac Oncol. 2017;12:1611–25. 28818606 10.1016/j.jtho.2017.08.002 PMC5659942

[5] Ou SI Nagasaka M. A catalog of 5’ fusion partners in ROS1-positive NSCLC circa 2020. JTO Clin Res Rep. 2020;1:100048. 34589944 10.1016/j.jtocrr.2020.100048 PMC8474457

[6] Uguen A Braekeleer MD. ROS1 fusions in cancer: a review. Future Oncol. 2016;12:1911–28. 27256160 10.2217/fon-2016-0050

[7] Shaw AT Ou SH Bang YJ. Crizotinib in ROS1-rearranged non-small-cell lung cancer. N Engl J Med. 2014;371:1963–71. 25264305 10.1056/NEJMoa1406766 PMC4264527

[8] Shaw AT Riely GJ Bang YJ. Crizotinib in ROS1-rearranged advanced non-small-cell lung cancer (NSCLC): updated results, including overall survival, from PROFILE 1001. Ann Oncol. 2019;30:1121–6. 30980071 10.1093/annonc/mdz131 PMC6637370

[9] Patil T Smith DE Bunn PA. The incidence of brain metastases in stage IV ROS1-rearranged non-small cell lung cancer and rate of central nervous system progression on crizotinib. J Thorac Oncol. 2018;13:1717–26. 29981925 10.1016/j.jtho.2018.07.001 PMC6204290

[10] Drilon A Siena S Dziadziuszko R. Entrectinib in ROS1 fusion-positive non-small-cell lung cancer: integrated analysis of three phase 1–2 trials. Lancet Oncol. 2020;21:261–70. 31838015 10.1016/S1470-2045(19)30690-4 PMC7811790

[11] Camidge DR Kim HR Ahn MJ. Brigatinib versus crizotinib in ALK-positive non-small-cell lung cancer. N Engl J Med. 2018;379:2027–39. 30280657 10.1056/NEJMoa1810171

[12] Shaw AT Kim DW Mehra R. Ceritinib in ALK-rearranged non-small-cell lung cancer. N Engl J Med. 2014;370:1189–97. 24670165 10.1056/NEJMoa1311107 PMC4079055

[13] Friboulet L Li N Katayama R. The ALK inhibitor ceritinib overcomes crizotinib resistance in non-small cell lung cancer. Cancer Discov. 2014;4:662–73. 24675041 10.1158/2159-8290.CD-13-0846 PMC4068971

[14] Lim SM Kim HR Lee J-S. Open-label, multicenter, phase II study of ceritinib in patients with non–small-cell lung cancer harboring ROS1 rearrangement. J Clin Oncol. 2017;35:2613–8. 28520527 10.1200/JCO.2016.71.3701

[15] Hirota T Muraki S Ieiri I. Clinical pharmacokinetics of anaplastic lymphoma kinase inhibitors in non-small-cell lung cancer. Clin Pharmacokinet. 2019;58:403–20. 29915924 10.1007/s40262-018-0689-7

[16] Zhu YC Zhang XG Lin XP. Clinicopathological features and clinical efficacy of crizotinib in Chinese patients with ROS1-positive non-small cell lung cancer. Oncol Lett. 2019;17:3466–74. 30867785 10.3892/ol.2019.9949 PMC6396181

[17] D’Angelo A Sobhani N Chapman R. Focus on ROS1-positive Non-Small Cell Lung Cancer (NSCLC): crizotinib, resistance mechanisms and the newer generation of targeted therapies. Cancers (Basel). 2020;12:3293. 33172113 10.3390/cancers12113293 PMC7694780

[18] Muller IB De Langen AJ Honeywell RJ. Overcoming crizotinib resistance in ALK-rearranged NSCLC with the second-generation ALK-inhibitor ceritinib. Expert Rev Anticancer Ther. 2016;16:147–57. 26654422 10.1586/14737140.2016.1131612

[19] Keddy C Shinde P Jones K. Resistance profile and structural modeling of next-generation ROS1 tyrosine kinase inhibitors. Mol Cancer Ther. 2022;21:336–46. 34907086 10.1158/1535-7163.MCT-21-0395 PMC8828706

[20] Wu Y-L Yang JC-H Kim D-W. Phase II study of crizotinib in east asian patients with ROS1-positive advanced non–small-cell lung cancer. J Clin Oncol. 2018;36:1405–11. 29596029 10.1200/JCO.2017.75.5587

[21] Remon J Pignataro D Novello S. Current treatment and future challenges in ROS1- and ALK-rearranged advanced non-small cell lung cancer. Cancer Treat Rev. 2021;95:102178. 33743408 10.1016/j.ctrv.2021.102178

[22] Hochmair MJ Fabikan H Illini O. Later-line treatment with lorlatinib in ALK- and ROS1-rearrangement-positive NSCLC: a retrospective, multicenter analysis. Pharmaceuticals (Basel). 2020;13:371. 33171712 10.3390/ph13110371 PMC7694976

[23] Hegde A Hong DS Behrang A. Activity of brigatinib in crizotinib and ceritinib-resistant ROS1- rearranged non-small-cell lung cancer. JCO Precis Oncol. 2019;3:PO.18.00267. 32775947 10.1200/PO.18.00267 PMC7410165

[24] Sun TY Niu X Chakraborty A. Lengthy progression-free survival and intracranial activity of cabozantinib in patients with crizotinib and ceritinib-resistant ROS1-positive non-small cell lung cancer. J Thorac Oncol. 2019;14:e21–4. 30217491 10.1016/j.jtho.2018.08.2030

[25] Shaw AT Bauer TM de Marinis F. First-line lorlatinib or crizotinib in advanced ALK-positive lung cancer. N Engl J Med. 2020;383:2018–29. 33207094 10.1056/NEJMoa2027187

[26] Zhang S Anjum R Squillace R. The Potent ALK inhibitor brigatinib (AP26113) overcomes mechanisms of resistance to first- and second-generation ALK inhibitors in preclinical models. Clin Cancer Res. 2016;22:5527–38. 27780853 10.1158/1078-0432.CCR-16-0569

[27] Davies KD Mahale S Astling DP. Resistance to ROS1 inhibition mediated by EGFR pathway activation in non-small cell lung cancer. PLoS One. 2013;8:e82236e82236. 24349229 10.1371/journal.pone.0082236 PMC3862576

[28] Roys A Chang X Liu Y. Resistance mechanisms and potent-targeted therapies of ROS1-positive lung cancer. Cancer Chemother Pharmacol. 2019;84:679–88. 31256210 10.1007/s00280-019-03902-6

[29] Facchinetti F Loriot Y Kuo MS. Crizotinib-resistant ROS1 mutations reveal a predictive kinase inhibitor sensitivity model for ROS1- and ALK-rearranged lung cancers. Clin Cancer Res. 2016;22:5983–91. 27401242 10.1158/1078-0432.CCR-16-0917

[30] Gainor JF Tseng D Yoda S. Patterns of metastatic spread and mechanisms of resistance to crizotinib in ROS1-positive non-small-cell lung cancer. JCO Precis Oncol. 2017;2017:PO.17.00063. 29333528 10.1200/PO.17.00063 PMC5766287

[31] Acquired resistance to crizotinib from a mutation in CD74–ROS1. N Engl J Med. 2013;369:1172–3. 24047073 10.1056/NEJMc1309091

[32] Chong CR Bahcall M Capelletti M. Identification of existing drugs that effectively target NTRK1 and ROS1 rearrangements in lung cancer. Clin Cancer Res. 2017;23:204–13. 27370605 10.1158/1078-0432.CCR-15-1601 PMC5203969

